# Impact of global smoking prevalence on mortality: a study across income groups

**DOI:** 10.1186/s12889-024-19336-6

**Published:** 2024-07-04

**Authors:** Roshinie De Silva, Disuri Silva, Lakindu Piumika, Isuri Abeysekera, Ruwan Jayathilaka, Lochana Rajamanthri, Colinie Wickramaarachchi

**Affiliations:** SLIIT Business School, Sri Lanka Institute of Information Technology, New Kandy Road, Malabe, Sri Lanka

**Keywords:** Mortality, Cardiovascular deaths, Lung cancer deaths, Respiratory deaths, Stroke deaths, Smoking prevalence, Smoking, Global smoking, Health

## Abstract

**Background:**

Smoking significantly contributes to the mortality rates worldwide, particularly in non-communicable and preventable diseases such as cardiovascular ailments, respiratory conditions, stroke, and lung cancer. This study aims to analyse the impact of smoking on global deaths, and its association with mortality across the main income groups.

**Methods:**

The comprehensive analysis spans 199 countries and territories from 1990 to 2019. The study categorises countries into four income groups: high income, upper middle income, lower middle income, and low income.

**Results:**

The findings underscore the profound impact of global tobacco smoking on mortality. Notably, cardiovascular disease mortality is notably affected in both upper-middle-income and high-income groups. Chronic respiratory disease mortality rates show a significant impact across all income groups. Moreover, stroke-related mortality is observed in the lower-middle, upper-middle, and high-income groups. These results highlight the pervasive influence of smoking prevalence on global mortality, affecting individuals across various socioeconomic levels.

**Conclusion:**

The study underscores the critical implications of smoking on mortality rates, particularly in high-income countries. It emphasises the urgency of targeted interventions in these regions to address the specific challenges posed by tobacco smoking on public health. Policy recommendations include implementing prohibitive measures extending to indoor public areas such as workplaces and public transportation services. Furthermore, allocating funds for research on tobacco and health, is imperative to ensure policymakers are consistently informed about emerging facts and trends in this complex domain.

**Supplementary Information:**

The online version contains supplementary material available at 10.1186/s12889-024-19336-6.

## Background

In recent years, the prevalence of smoking has emerged as a significant contributor to global mortality rates. By 2020, smoking was reported to have contributed to 22.3% of worldwide deaths annually due to excessive tobacco use [1]. Cardiovascular diseases are known to be the leading cause of death globally posing a threat to 182 countries and responsible for 90% of chronic respiratory deaths [2–5]. Despite economic differences, the majority of smokers succumb to cardiovascular disease [6–8], highlighting the severe impact of tobacco usage on one in ten adults [6, 7] and their quality of life [9].

However, these diseases are more prevalent in Low Income (LI) and Lower Middle Income (LMI) countries [4, 10–12], accounting for a significant share of all cardiovascular mortality rates globally [13] and leading to high economic and social burden [14, 15]. Studies conducted in North Africa, the Middle East and South Africa reveal that chronic respiratory deaths occur predominantly due to smoking [16, 17]. Results show that women have a lower prevalence of smoking than men, particularly among youth and adults [7]. Respiratory diseases, tuberculosis, and ischaemic heart diseases act as leading risk factors for smoking, further increasing the risk of stroke [18]. In Indonesia, the majority of deaths are due to cardiovascular diseases, with a significant proportion related to smoking [19], attributed to unhealthy lifestyles [20]. Earlier econometric studies have concluded that increased smoking over the years has led to rises in cardiovascular diseases and premature deaths [21, 22].

Furthermore, studies incorporating both Cardiovascular Disease Death Rates (CDDR) and Stroke Death Rates (SDR) revealed that current smokers display symptoms of both stroke and vascular-related symptoms irrespective of their educational level and financial state [23, 24]. Another study investigated more than 52 countries in the LMI and Upper Middle Income (UMI) brackets in relation to Chronic Respiratory Disease Death Rates (CRDDR), revealing that over half of the country’s population shows symptoms of respiratory diseases and addicted to tobacco-related products [25, 26]. The study predicts that by 2030, 80% of all global deaths will be due to chronic respiratory diseases among both income groups. These studies often focus on specific time periods, resulting in gaps in literature.

Additionally, concerning lung cancer-related deaths, researchers identified high-risk increase in smoking in LMI countries, emphasising the importance of early detection to support preventive measures [27]. Statistical methodologies used in previous studies are often outdated. Ethnic-related studies in China revealed that certain ethnic populations [28–31] such as the ‘Han’ have a lower prevalence of cardiovascular diseases despite high smoking prevalence. However, smoking prevalence among the ‘Han’ remains high. In Mexico, the highest risk from smoking is associated with cardiovascular diseases and related deaths [8], although these studies have not incorporated more than two dependent variables.

Regarding CRDDR, a study found that smoking is widespread among Chinese men in the middle-income category, with similar trends observed in East Asian and Pacific region countries with a high tendency towards respiratory disease-related deaths [32]. However, many studies interpret mortality rate results compared to vascular and respiratory diseases through simplistic data analysis. Empirical gender-related studies have highlighted higher chronic respiratory disease cases and smoking incidences in males than females [33].

Concerning SDR, a study conducted in Cuba, portrays 786.6 deaths per 100,000 people due to stroke-related deaths [34]. Stroke-related deaths have significantly increased among male smokers. Smoking cessation among the Chinese does not appear to reduce risk of stroke deaths [35]. Finally, Lung Cancer Death Rates (LCDR) are highlighted in a study conducted in Shanghai, indicating a change in serum miRNAs as potential biomarkers for different cancers, including lung cancer, associated with cigarette smoking [36]. Another study identified the prevalence of lung cancers among smokers in Shanghai from 2016 to 2017 showing an increased proportion of lung cancer deaths due to excessive smoking [37].

Results of studies considering ‘time’ and the risk of Cardiovascular Diseases (CVDs) due to smoking prevalence indicate a higher susceptibility to CVDs among the young population and women [38, 39]. Even smoking one cigarette per day could significantly increase the risk of cardiovascular diseases, serving as a vital independent risk factor for cardiovascular disease-related deaths [40, 41]. Researchers concluded that smokers have a higher risk of cardiovascular diseases compared to non-smokers [42].

Moreover, a study on chronic respiratory diseases and lung cancer identified patients continued smoking habits, even after as the main cause [43]. Those diagnosed with these conditions showed a lower rate of engaging with smoking cessation agents to prevent disease severity.

A strong nexus between stroke and smoking in middle-aged men in Norway indicates increased risk of cardiovascular diseases and cancer [44]. A study in New Zealand identified a relatively high risk of acute stroke associated with exposure to environmental tobacco smoke and passive smoking [45]. Compared to non-smokers, there is a higher risk of SDR in men and women who smoke one cigarette a day. Gender differences are thoroughly assessed interpreting the findings of both research studies.

The risk of lung cancer increases with the number of cigarettes smoked daily [46]. Studies form Finland [47], Japan [48] and the US [49] reviewed the relationship between smoking prevalence and the risk of lung cancer and respiratory diseases. They concluded that there is a strong association between smoking prevalence and the occurrence of lung cancer and respiratory diseases. Smoking cessation is recommended tom minimise lung cancer risk.

However, in high-income countries a strong association exists between smoking prevalence and CDDR, CRDDR, SDR, and lung cancer death rates. High-income countries may have better access to smoking prevalence data compared to UMI, LMI, and LI countries.

Despite systematic efforts to alleviate death rates through nicotine treatment, trigger avoidance, physical activities, and preventative measures, outcomes might be slow. Empirical studies have consistently associated cigarette smoking with a significantly higher risk of dying from chronic respiratory diseases [33, 50]. However, these studies often focus solely on chronic respiratory and cardiovascular death rates and are limited to specific countries [24, 51]. A comprehensive global study capturing the impact of Global Tobacco Smoking Prevalence (GSP) on CDDR, CRDDR, SDR, and LCDR over time, considering different income levels in 199 countries, remains unexplored. Hence, there is a visible gap in the existing literature that requires attention.

This paper aims to determine the impact of global smoking prevalence on worldwide mortality rates due to cardiovascular diseases, chronic respiratory diseases, stroke, and lung cancer. The study seeks to contribute to the existing literature in three ways:

First, this research fills a gap by conducting, to the authors’ knowledge, the first quantitative study incorporating four dependent variables in a global mortality analysis, cross-referencing with the GSP as the independent variable, with all variables age-standardised.

Second, the study methodology differs from previous literature by employing a panel data regression model covering thirty years from 1990 to 2019 for 199 countries, categorised into four primary income levels.

Third, the research aims to explore and prioritise age-standardised death rates for cardiovascular diseases globally, comparing countries across income groups and offering diverse perspectives on the findings.

## Methods

This section presents the samples and observations derived from data spanning from 1990 to 2019, covering a 30-year period for 199 countries, excluding five countries not assigned an income group by the World Bank. Age-standardised rates were utilised in this study’s analysis to ensure that the statistical results for the income groups were not influenced by variations in age distributions across different countries. The dataset is divided into four income group classifications as defined by the World Bank [52]. The independent variable in this study is GSP, and the dependent variables are CDDR, CRDDR, SDR, and LCDR. The data file used in this study is attached in S1 Appendix.

### Data

This study uses secondary data sources with the data file presented in S1 Appendix. Data were collected from three databases to obtain health outcome data for the five variables, detailed in Table 1.

**Table 1:** Data sources and variables

Variable		Definitions	Source	
GSP	Global Smoking Prevalence per 100,000 people.			IHME, Global Burden of Disease (2019) https://ghdx.healthdata.org/record/ihme-data/gbd-2019-smoking-tobacco-use-prevalence-1990-2019
CDDR	The annual number of deaths from cardiovascular diseases per 100,000 people.			Our World in Data database (2021) https://ourworldindata.org/grapher/cardiovascular-disease-death-rates
CRDDR	The annual number of deaths from chronic respiratory diseases per 100,000 people.			Our World in Data database (2021) https://ourworldindata.org/grapher/respiratory-disease-death-rate
SDR	Strokes Deaths per 100,000 individuals.			Our World in Data database (2021) https://ourworldindata.org/grapher/stroke-death-rates
LCDR	Several lung, bronchus, and trachea cancer deaths per 100,000 people.			WHO mortality Database (2022) https://platform.who.int/mortality

### Statistical analysis

**Fig. 1:**
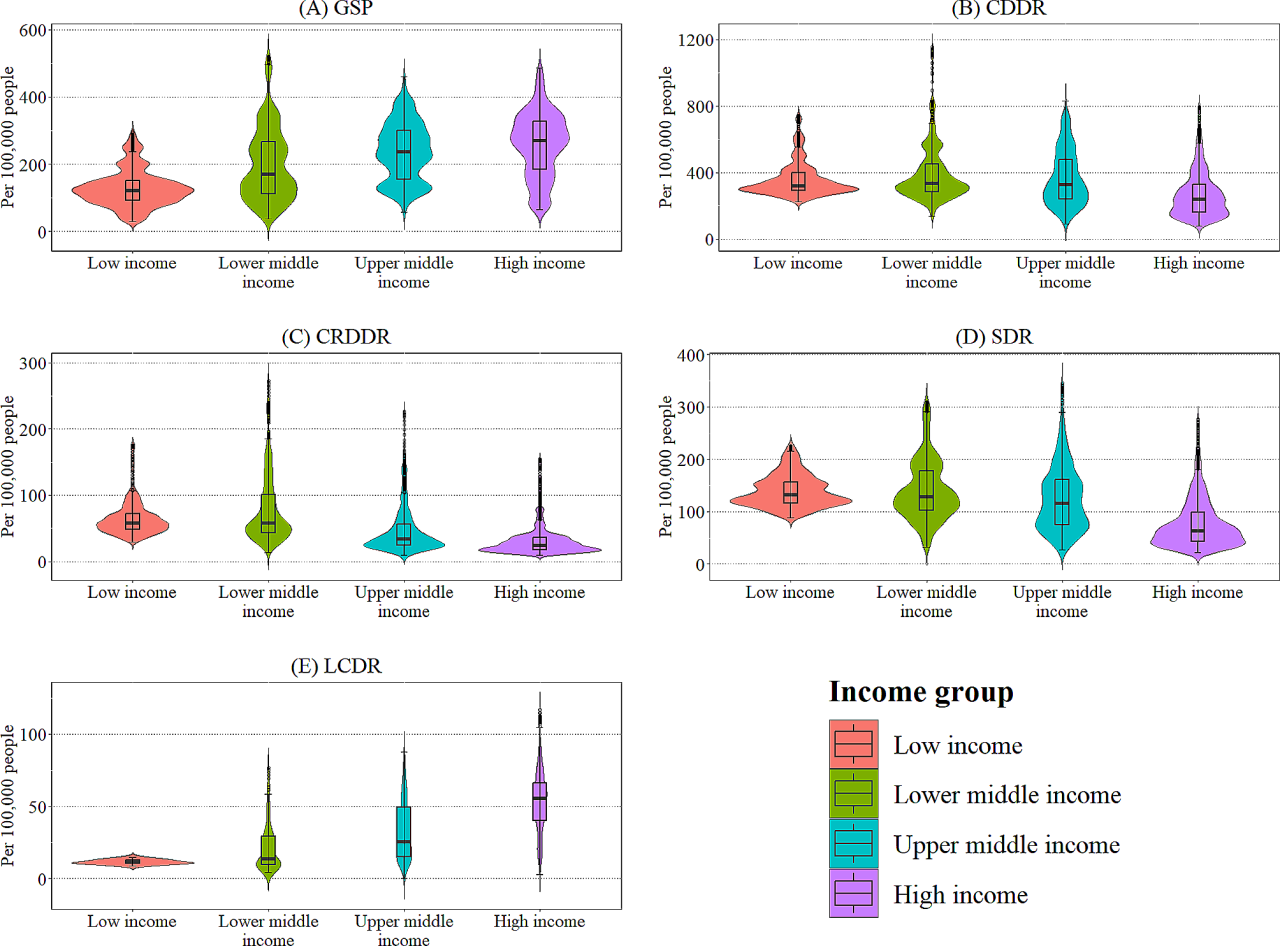
Summary of descriptive statistics illustrated as a violin plot by critical variables Source: Authors’ illustrations based on data

Descriptive statistics were used to summarise and explore the data. Figure 1 presents a summary of descriptive statistics using a violin plot combined with a box plot, according to the data in Table 2. The violin plot provides a comprehensive visualisation of data density and range, including the five-number summary and outliers graphically represented through the box plot [53].

**Table 2:** Summary of descriptive statistics for the critical variables by income group

Income Group	Variables					
		GSP	CDDR	CRDDR	SDR	LCDR
Low Income	Obs.	840	840	840	840	13
	Mean	129.127	360.194	64.044	139.33	11.603
	SD	53.819	104.694	24.427	30.565	1.7385
	Min	30.633	225.24	29.75	87.75	8.84
	Max	297.085	741.17	175.44	225.35	14.9
Lower Middle Income	Obs.	1,590	1,590	1,590	1,590	217
	Mean	198.987	389.057	78.470	140.354	22.084
	SD	106.237	160.289	50.754	56.594	16.886
	Min	38.052	137.38	13.41	31.46	4.35
	Max	521.122	1156.2	273.02	310.23	76.72
Upper Middle Income	Obs.	1,590	1,590	1,590	1,590	930
	Mean	236.570	366.746	46.276	124.913	32.501
	SD	85.737	162.023	33.920	61.709	20.702
	Min	56.856	88.61	9.32	26.47	0
	Max	460.705	830.9	226.43	345.53	87.38
High Income	Obs.	1,950	1,950	1,950	1,950	1,349
	Mean	258.022	268.986	30.867	77.570	53.914
	SD	95.871	138.159	21.814	47.898	20.928
	Min	65.436	77.01	9.92	21.57	2.97
	Max	486.312	792.91	155.03	276.91	116.55

Note: Obs. represents the number of observations, SD represents the standard deviation, Min represents the minimum value, and Max represents the maximum value

The panel regression model for time variation data to identify the impact of GSP on CDDR, CRDDR, SDR and LCDR is given below. Four separate equations are regressed for the $i$^th^ cross section (income group) units at time t (years), with ε accounting for standard errors:

1 $${CDDR}_{it}={\alpha }_{0}+{\alpha }_{1}{GSP}_{it}+{\epsilon }_{it}$$

Where ${\alpha }_{1}$ represents the estimated increase in CDDR per 100,000 people over the change in global smoking prevalence and ${\alpha }_{0}$ is the intercept.

2 $${CRDDR}_{it}={\beta }_{0}+{\beta }_{1}{GSP}_{it}+{\epsilon }_{it}$$

Similarly, ${\beta }_{1}$ represents the positive increase in CRDDR over the change in global smoking prevalence, with ${\beta }_{0}$ as the intercept.

3 $${SDR}_{it}={\gamma }_{0}+{\gamma }_{1}{GSP}_{it}+{\epsilon }_{it}$$

In this equation, ${\gamma }_{1}$ associates SDR with smoking prevalence, while ${\gamma }_{0}$ is the intercept.

4 $${LCDR}_{it}={\delta }_{0}+{\delta }_{1}{GSP}_{it}+{\epsilon }_{it}$$

Here, ${\delta }_{1}$ reflects the effect of GSP on LCDR, with ${\delta }_{0}$ as the intercept. Through the incorporation of the co-efficient, α_1_, β_1_, γ_1_ and δ_1_, this study attempts to test the hypothesis that the independent variable significantly affects the dependent variables, offering a thorough insight into the impact on each dependent variable through the panel regression model. For the regression models concerning CDDR, CRDDR, SDR, and LCDR, we formulated the following hypotheses:

- CDDR [${\alpha }_{1}$> 0]: Higher GSP levels would be linked to higher rates of chronic disease-related mortality. This hypothesis was grounded in literature suggesting that increased economic development improves healthcare infrastructure and overall public health.
- CRDDR [${\beta }_{1}$> 0]: Higher GSP would impact higher mortality rates from chronic respiratory diseases, supported by previous research indicating the association of economic development with improved respiratory health outcomes.
- SDR [${{\gamma }_{1}}_{1}$> 0]: Higher GSP levels would influence overall higher mortality rates across various causes, based on the premise that economic development facilitates improvements in healthcare provision and disease prevention measures.
- LCDR [${\delta }_{1}$> 0]: Higher GSP would result in higher mortality rates from lung cancer, informed by studies that highlight the relationship between economic development, lifestyle factors, and access to healthcare resources, all of which influence lung cancer incidence and mortality rates.

Moreover, this study computes three potentially stabilised models in consideration to the literature such as Pooled Ordinary Least Squares (POLS) to focus on dependencies between individuals [54], the Fixed Effects (FE) model to determine individual unobserved effects and the Random Effects (RE) model [55, 56] to focus on both dependencies between and within individuals [57] in analysing the balanced panel data regression. On the other hand, the specification tests F-Test, Breusch-Pagan [58, 59] and Hausman test [60, 61] were applied to select the appropriate estimator from the results generated. Furthermore, the issue of multicollinearity is exempted as this study comprises only one independent variable, aligning with the methodology [62]. Finally, statistical data analysis was conducted using Stata and R Studio software.

## Results

The descriptive statistics for the critical variables used in this study are summarized in Table 2. For example, the highest GSP value was 521 per 100,000 people in Kiribati in 2004, while the highest mean GSP was 258 per 100,000 people in the high-income (HI) group. The LMI group represents the highest mean values for CDDR, CRDDR, and SDR. The most significant occurrence of CDDR was 1156 per 100,000 people in Uzbekistan in 2005. The highest mean CRDDR was observed in the LMI group, while the highest mean SDR was 140 per 100,000 people in the LMI group.

Figure 1 portrays violin plot diagrams for five variables categorised into four major income groups. This plot highlights the relationship between income groups and global deaths per 100,000 people. The box plot elements show the lower and median fatalities in the HI group for SDR and LCDR, respectively. Both CDDR and CRDDR have a long-tail distribution for the LMI group. LCDR does not show wide area dispersions for all income levels as no frequent values are exposed. In the case of LI countries, all variables except LCDR show higher probabilities. Narrow spread plots are visible in CRDDR and LCDR for the LMI group. GSP and SDR variables visualise wider dispersions compared to the other variables. The data regarding the independent variable show multiple mode values under all four income groups. In comparison, the variables GSP and SDR portray higher dispersions.

Further elaborating on the plots, it is evident that CDDR and CRDDR have the highest outliers for the LMI group compared to the other variables and stratums. On the other hand, the UMI group shows no outliers for the variables GSP, CDDR and LCDR. Moreover, GSP has no outliers in the HI group, and LCDR has zero outliers for the LI stratum. The side flip of this plot depicts a mixture of a histogram and a density plot. The box plot diagram is attached to S2 Appendix for further clarification..

**Fig. 2:**
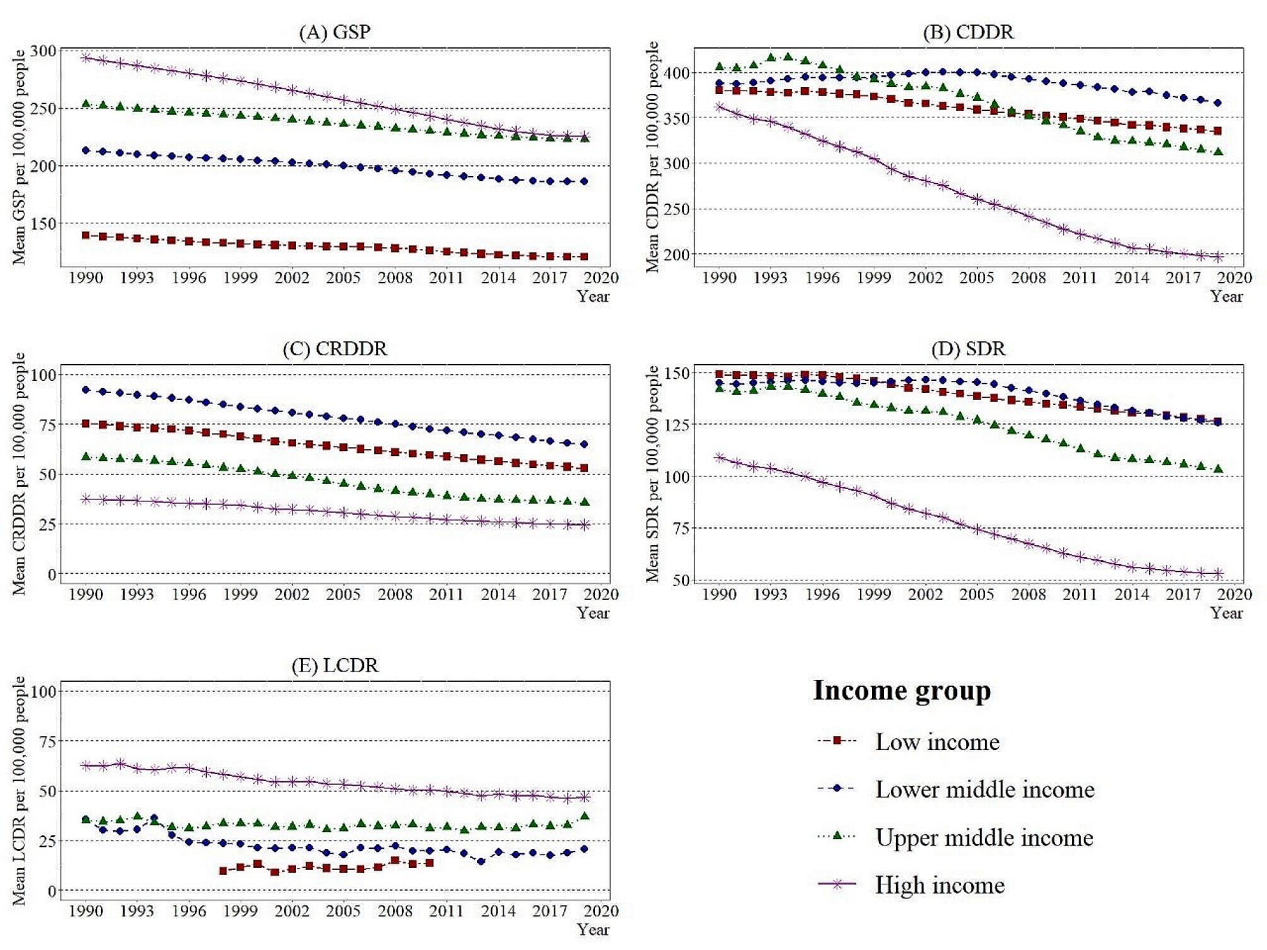
Income group-wise averaged variables from 1990–2019 Source: Authors’ illustrations based on data

Figure 2 depicts line charts symbolising the mean disparity over the years from 1990 to 2019, reflecting the income groups in the research study. Examination of GSP, presented in Fig. 2A, shows that the LI group indicated the lowest rate and the HI group the highest rate of mean GSP per 100,000 people in 1990. A continuous decline of 0.44%, 0.49%, 0.47% and 0.91% on average per year can be observed for LI, LMI, UMI, and HI countries, respectively, resulting in the lines for the HI and UMI groups covering around the year 2016.

Likewise, Fig. 2B represents the mean variations of CDDR per 100,000 people. Accordingly, the mean death rates from cardiovascular diseases for all four income groups were 350–410 per 100,000 people in 1990. The line plotted for HI and LI countries shows a decline of 2.08% and 0.44% on average per year, respectively. The UMI countries ranked first from 1990 to 1997 and fell to third place in 2009, preceded by the LMI and LI groups, respectively. The UMI group has shown a drop of 23.11% overall from 1990 to 2019, intercepting the LMI and LI group lines in 1998 and 2007, respectively.

Figure 2C shows a drop of 1.20%, 1.21%, 1.71% and 1.47% per year in LI, LMI, UMI and HI groups for the mean CRDDR per 100,000 people. At the same time, LI and HI groups continue to have the highest and the lowest mean deaths per 100,000 people, respectively.

Figure 2D portrays a drastic decline of 51.46% for the mean SDR per 100,000 people in the HI group. Furthermore, the HI group, which had a difference of around 33 mean SDR per 100,000 people compared to the other three income groups in 1990, increased the contrast to at least 50 per 100,000 people by 2019. The LMI group, which was in second place in 1990, surpassed the LI group by 2000 to take first place. Subsequently, the LMI group shows a slight decrease from 2005 and intersects with LI group by 2015.

Figure 2E presents the line plot for mean LCDR per 100,000 people according to income groups. The HI group remains the highest from 1990 to 2019, with a decline of 0.98% on average per year. Unfortunately, the line plot of the LI group does not provide a factual illustration due to insufficient data.

### Panel regression analysis

Results from the panel data regression conducted for the LI and LMI groups are portrayed in Table 3. The outcome suggests that for the LI group, the RE model was suitable for the variables CDDR, CRDDR and SDR, except for LCDR due to insufficient data. Likewise, for the LMI group, the RE model is suggested for all four dependent variables. For the LI group, the RE model estimates that GSP has a positive influence on CRDDR at a 5% significance level, whereby when GSP increases by one per 100,000 people, CRDDR has shown an increase across time by an average of 18%. For the LMI group, the RE estimates show a positive influence of GSP on CRDDR at a 1% significance level, with an increase of GSP by 1 per 100,000 people affecting CRDDR by 19% on average. Furthermore, GSP indicates a positive and statistically significant effect of 10% on SDR, whereby SDR increases by 17% whenever GSP increase by one per 100,000 people.

**Table 3:** Fixed effect and random effect estimates

		**Low Income**											
		CDDR				CRDDR			SDR			LCDR	
GSP		0.3161		0.313		0.1813**	0.1905**		0.2319	0.2499		*a*	-0.137
		-0.418		-0.422		-0.087	-0.092		-0.144	-0.164		*a*	*a*
Constant		319.3792***		319.7815***		40.6292**	39.4493**		109.3859***	107.0604***		*a*	48.1712
		-61.911		-54.469		-12.562	-11.819		-20.879	-21.129		*a*	*a*
R^2^	Within	0.018				0.0753			0.054			0.2511	
	Between	0.0392				0.0201			0.0325			a	
	Overall	0.0375				0.0229			0.0328			0.2511	
No. of years / No. of countries							30 / 28					13 / 01	
N		840										13	
F-test / LM test			356.97*** /1,0301.29***			226.73*** / 9,324.46***			132.15*** / 7,885.75***			*a*	
Hausman test		0.03				2.18			2.09			*a*	
		**Lower Middle Income**											
GSP		0.2295		0.2085		0.1917***	0.1901**		0.1708*	0.1661**		0.0904	0.0831
		-0.198		-0.213		-0.052	-0.054		-0.065	-0.068		-0.062	-0.072
Constant		343.3921***		347.5593***		40.3223***	40.6488***		106.368***	107.3117***		4.8591	5.7946
		-39.454		-42.339		-10.792	-10.777		-12.32	-13.613		-10.83	-14.183
R^2^	Within	0.0101				0.1284			0.0492			0.0882	
	Between	0.1202				0.2278			0.1937			0.4768	
	Overall	0.1099				0.2225			0.1814			0.6381	
No. of years / No. of countries							30 / 53						
N							1,590					217	
F-test / LM test			305.07***/1,8962.18***			488.78***/2,0403.78***			282.42***/1,8731.42***			46.34***/1,065.73***	
Hausman test		2.28				0.38			0.87			0.69	

Note: The asterisks, *, ** and *** indicate 10%, 5% and 1% significance levels, respectively robust standard errors in parentheses. FE and RE represent the Fixed Effect and Random Effect, respectively. N shows the number of observations. The hypothesis for F-test is Ho: Accepting POLS and Ha: Accepting FE Model, the hypothesis for the Breusch-Pagan test is Ho: Accepting POLS and Ha: Accepting RE Model and the hypothesis for the Hausman test are Ho: Accepting RE Model and Ha: Accepting FE Model. ‘a’ represents the values that were not able to obtain due to missing data cases

The statistical results for the UMI and HI groups are represented in Table 4. For the UMI group, the RE model was deemed appropriate for the variables CDDR, CRDDR, and SDR, but not for LCDR. Conversely, for the HI group, the RE model was more suitable for CRDDR and LCDR, while the FE model was preferred for CDDR and SDR. Consequently, both the FE and RE models were separately applied to all dependent variables within the UMI and HI groups. The RE estimates for the UMI group indicated that GSP has a highly significant positive effect on CDDR and SDR at the 1% level, suggesting that an increase in GSP by one per 100,000 people will result in an average increase of 72% in CDDR and 27% in SDR over time.

**Table 4:** Fixed effect and random effect estimates

		**Upper Middle Income**										
		CDDR			CRDDR			SDR			LCDR	
GSP		0.7178***	0.7062***		0.1325**	0.1377**		0.2676***	0.2591***		0.0357	0.0168
		-0.179	-0.186		-0.054	-0.057		-0.063	-0.066		-0.022	-0.022
Constant		196.9241***	199.6705***		14.9385	13.6982		61.6160***	63.6094***		24.3687***	28.594***
		-42.617	-44.081		-11.47	-13.51		-15.837	-15.531		-5.754	-5.179
R^2^	Within	0.0917			0.0572			0.0821			0.0042	
	Between	0.2376			0.0267			0.299			0.6007	
	Overall	0.2215			0.0288			0.2734			0.5533	
No. of years		30										
No. of countries		53									40	
N		1,590									930	
F-test / LM test		210.49*** / 1,7523.80***			177.98***/1,6673.14***			184.34***/ 1,6834.74***			90.57*** / 4,691.75***	
Hausman test		0.71			1.97			2.19			63.13***	
		**High Income**										
GSP		1.2120***	1.2619***		0.0834***	0.0841***		0.3801***	0.3998***		0.1288***	0.1277***
		-0.107	-0.116		-0.02	-0.02		-0.047	-0.052		-0.021	-0.021
Constant		-43.727	-56.6249*		9.3587**	9.1595*		-20.499	-25.5747*		18.0866**	19.3567**
		-32.094	-29.832		-4.593	-5.219		-12.552	-13.449		-5.425	-5.786
R^2^	Within	0.3988			0.1416			0.2859			0.3254	
	Between	0.0016			0.0628			0.0233			0.423	
	Overall	0.015			0.0692			0.0433			0.3911	
No. of years / No. of countries						30 / 65						
N						1,950					1,349	
F-test / LM test		218.88*** /1,8881.68***			297.96***/ 2,3194.19***			144.93***/1,7515.66***			168.39*** /1,2860.79***	
Hausman test		105.52***			0.91			40.14***			1.62	

Note: The asterisks, *, ** and *** indicate 10%, 5% and 1% significance level, respectively. Robust standard errors in parentheses. FE and RE represent the Fixed Effect and Random Effect, respectively. N shows the number of observations. The hypothesis for F-test is Ho: Accepting POLS and Ha: Accepting FE Model, the hypothesis for the Breusch-Pagan test is Ho: Accepting POLS and Ha: Accepting RE Model and the hypothesis for the Hausman test are Ho: Accepting RE Model and Ha: Accepting FE Model

It also shows a statistically significant positive effect for CRDDR at 5%, with GSP increasing CRDDR by 13%. Likewise, the estimates for the HI group imply that GSP positively influences CDDR, CRDDR, SDR, and LCDR at a 1% significance level. The model coefficients for CDDR, CRDDR, SDR, and LCDR show increases of 126%, 8%, 40% and 13% on average, respectively.

## Discussion

In examining the LI group, it is evident that GSP has a positive but insignificant impact on CDDR and SDR variables. However, a contradictory finding revealed that tobacco use was linked to a reduced risk of cardiovascular-related diseases and was identified as the most crucial risk factor for stroke [6]. Moreover, the study indicated that GSP influences death rates from chronic respiratory diseases only within the LI group. Additionally, smoking was found to be a primary risk factor for chronic respiratory diseases, making CRDDR the leading cause of mortality in the countries studied [16]. Due to limited data on the LCDR variable, relevant results could not be obtained for the LI group. Nonetheless, it was found that higher smoking rates correlate with an increased mortality burden from lung cancer [14].

For the LMI group, the investigation suggested that despite a positive relationship between GSP and CDDR, the effect is not significant. Supporting this, the study discovered that while there is no significant relationship between tobacco smoking and cardiovascular disease deaths, smoking does increase the risk of death from ischemic heart diseases [12]. Furthermore, the study showed that GSP significantly impacts only CRDDR and SDR. Current smokers had a higher risk of all-cause mortality, including deaths from cardiovascular diseases and stroke, compared to non-smokers [19].

The analysis in the UMI stratum, revealed that CDDR, CRDDR, and SDR significantly impact GSP. There is a positive relationship between deaths caused by stroke and daily smoking prevalence among both females and males [35]. This finding aligns with existing literature, which indicates that smoking prevalence among both genders contributes significantly to mortality rates from cardiovascular diseases and stroke [7, 33]. Studies have consistently shown a significant association between smoking status and cardiovascular mortality rates [63]. Previous research suggests that although smoking rates among males have historically been higher, the prevalence of smoking among females has been increasing, leading to a narrowing gender gap in smoking-related mortality [34, 38]. Our results underscore the importance of considering gender-specific factors in mortality analysis, as smoking behaviours and their associated health risks may vary between genders. For instance, while males may have higher overall smoking rates, females may be more susceptible to certain smoking-related health outcomes, such as lung cancer [44]. However, our study was limited by the unavailability of gender-specific data, preventing us from conducting a gender-stratified analysis to explore potential differences in smoking prevalence and its impact on mortality rates between males and females. At the HI level, the research showed that GSP substantially impacts CDDR, CRDDR, SDR, and LCDR, with a significant association between smoking status and cardiovascular mortality rates. Supported studies explicitly show a significant association between smoking status and cardiovascular mortality rates [40, 64].

Vertical analysis of the results, comparing the RE and FE coefficients for the UMI and HI groups by the dependent variable CDDR, suggests that GSP has a more significant effect on CDDR for HI countries than UMI countries. Similarly, RE coefficients for CRDDR across all four income groups imply that GSP has the highest impact on CRDDR in LMI countries and the lowest impact in HI countries. In the context of SDR, it is more affected by GSP in HI countries and least affected in LMI countries. Within the LMI group, CRDDR and SDR are more significantly affected by GSP, while in UMI countries, CDDR experiences the highest impact from GSP. In HI countries, CDDR, SDR, LCDR, and CRDDR experience varying degrees of impact from GSP, with CRDDR being the most affected in LI and LMI groups from 1990 to 2019, but in contradiction, almost all income groups show a high tendency in CRDDR [65].

Previous studies have examined the impact of GSP on CVD, chronic respiratory diseases, stroke, and lung cancer, considering factors other than a country’s income level. A study on the burden of CVD attributable to smoking during the same period found a reduction in CVD-related deaths from 1990 to 2019, with the lowest mortality rates in high socio-economic regions in 2019 [66]. Similarly, research on risk factors for chronic respiratory diseases and cancer from 1990 to 2019 supports that smoking remains a high-risk factor for these health issues despite lower incident rates [67, 68].

## Conclusion

Despite the extensive literature on global smoking prevalence and death rates, this study stands out by examining a combination of quadruple variables: CDDR, CRDDR, SDR, and LCDR across different income strata, rendering it unique. Tobacco smoking prevalence correlates directly with increased global deaths from cardiovascular diseases, respiratory diseases, stroke, and lung cancer among populations worldwide, primarily segmented by four income levels. However, the violin plot demonstrates minimal variations in LCDR, contrasting with noticeable variations in GSP and SDR across all income groups. A line graph illustrates a significant decline in mean death rates, particularly in the HI stratum from 1990 to 2019, with fluctuations in death rates among other income groups. Regression results indicate that a higher proportion of smoking prevalence deaths is attributable to cardiovascular diseases, particularly in the HI group. Variations are also observed in age-standardised prevalence death rates across different income levels and dependent variables, with chronic respiratory diseases and stroke deaths being predominant in UMI and LMI groups, respectively.

Nonetheless, this study has limitations, including the inability to consider data from 2020 to 2022 due to unavailability, lack of data on the LCDR variable, and reliance solely on deaths caused by specific diseases due to data constraints. However, it underscores the impact of global tobacco smoking on death rates from cardiovascular diseases, chronic respiratory diseases, stroke, and lung cancer.

Moreover, a pertinent limitation of this analysis is the absence of consideration towards the aspect of gender due to the insufficient availability of secondary data. As a consequence of this restriction, this study falls short in analysing the differences in the impact of GSP on mortality with respect to gender. By examining factors such as the age at which a person starts smoking regularly, gender differences in the duration and daily intake of smoking, and variations in environmental and occupational exposures related to age and gender, the study could offer a clearer understanding of how GSP affects mortality.

As a factor influencing CDDR, CRDDR, SDR, and LCDR rates, the socioeconomic burden of GSP is substantial, necessitating pragmatic policy implications. Implementing higher taxes on tobacco products and stringent regulations can effectively reduce GSP, especially among vulnerable groups like the youth. Additionally, revenue from increased tobacco taxes can be reinvested by governments into healthcare costs for treating tobacco-related illnesses.

Future research directions could focus on addressing critical gaps in understanding socioeconomic influences on health outcomes. Refining deprivation indices and studying their applicability across diverse populations could enhance health assessments [69, 70]. Studies could also investigate gender differences in the impact of smoking on mortality rates and track the effectiveness of health interventions over time, particularly in mitigating health disparities among low-income groups. Comparative studies across countries could shed light on how evolving socioeconomic conditions and health behaviours affect cardiovascular risk globally. Furthermore, future research could evaluate the efficacy of tobacco control programs across different socioeconomic strata to determine the most effective approaches to reduce tobacco-related mortality.

Moreover, longitudinal studies can track the impact of socioeconomic status cardiovascular risk factors and other diseases, assessing the effectiveness of health interventions in reducing disparities among low-income groups over the time [71]. Additionally, comparative data from studies conducted across different countries can analyse how evolving socioeconomic conditions and health behaviours influence cardiovascular risk in various global contexts [72]. Lastly, future research should investigate the efficacy of tobacco control programs across different socioeconomic strata, aiming to identify the most effective approaches to reducing tobacco-related mortality across diverse demographic segments [73].

Furthermore, awareness regarding the dangers of smoking and the benefits of quitting can be highlighted through investments in public education campaigns targeted primarily at towards those who are more vulnerable to the adverse health effects of smoking. To safeguard the health of non-smokers and deter smoking habits, the implementation of smoke-free policies is highly recommended, including smoking bans in public indoor places such as workplaces and public transport services.

Lastly, by investing in tobacco and health related research, policymakers can stay updated and informed about timely facts and trends, enabling them to generate new policies and health initiatives worldwide more practically and conveniently.

### Electronic supplementary material

Below is the link to the electronic supplementary material.

**Supplementary Material 1: S1 Appendix.** Data Set

**Supplementary Material 2: S2 Appendix.** Summary of descriptive statistics through a Boxplot diagram

## Data Availability

The datasets generated and analysed during the current study are publicly available at Our World in Data database: https://ourworldindata.org/ and Institute for Health Metrics and Evaluation (IHME) database: https://www.healthdata.org/.
