# Supplementary figures and images for: Impact of global smoking prevalence on mortality: a study across income groups
Source: BMC Public Health. 2024 Jul 4;24:1786. doi: 10.1186/s12889-024-19336-6 (PMC11225136; doi:10.1186/s12889-024-19336-6)

**
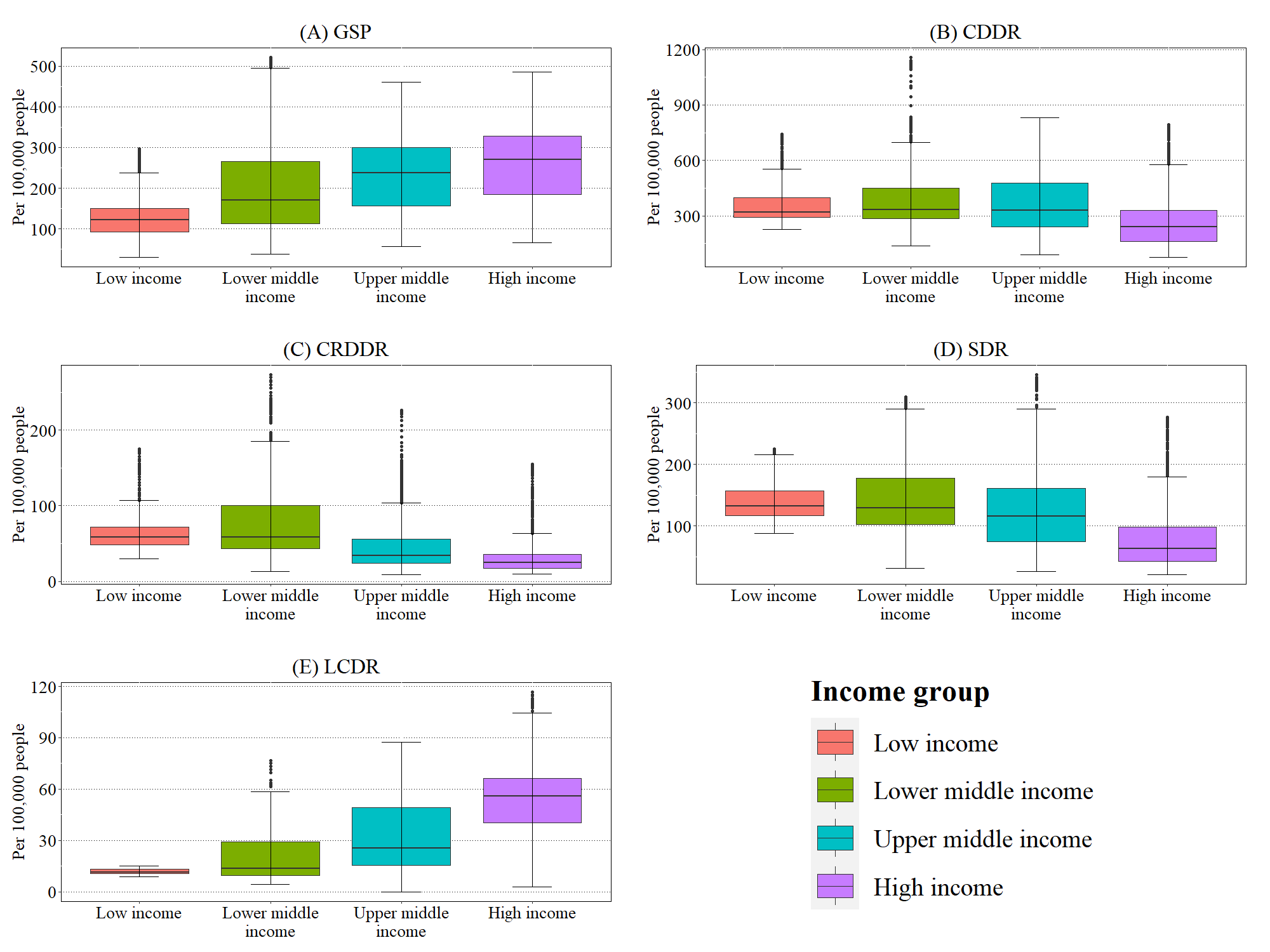
S2 Appendix: Summary of descriptive statistics through a Boxplot diagram**

Supplement: Supplementary file 2 — Supplementary Material 2: S2 Appendix. Summary of descriptive statistics through a Boxplot diagram [file 12889_2024_19336_MOESM2_ESM.docx]
